# Differences in Adiposity Profile and Body Fat Distribution between Forwards and Backs in Sub-Elite Spanish Female Rugby Union Players

**DOI:** 10.3390/jcm10235713

**Published:** 2021-12-06

**Authors:** Dolores Escrivá, Jordi Caplliure-Llopis, Inmaculada Benet, Gonzalo Mariscal, Juan Vicente Mampel, Carlos Barrios

**Affiliations:** 1 Intensive Care Unit, La Fe Polytechnic and University Hospital, 46026 Valencia, Spain; descpei@gmail.com; 2 Institute for Research on Musculoskeletal Disorders, School of Medicine, Valencia Catholic University, 46001 Valencia, Spain; jordi.kaliu@hotmail.com (J.C.-L.); inmabenet@yahoo.es (I.B.); gonzalo.mariscal@mail.ucv.es (G.M.); 3 Primary Health Care Services, La Ribera University Hospital, 46600 Alzira, Spain; 4 Surgical Emergency Facilities, Valencia University Hospital, 46010 Valencia, Spain; 5 Department of Physiotherapy, Catholic University of Valencia San Vicente Mártir, 46001 Valencia, Spain; juan.vicente@ucv.es

**Keywords:** women’s rugby, anthropometry, body composition, adiposity, somatotype

## Abstract

The purpose of this study was to analyze the adiposity profile and the body fat distribution in 56 sub-elite female rugby union players involved in the Spanish National Women’s Rugby Union Championships. The participants included in this study, which was the first to analyze sub-elite players, show thinner skinfolds, lower fat mass, and lesser fat percentage than previously reported for elite female rugby union players. Forwards were heavier and had higher body mass index (BMI) and fat mass, thicker skinfolds, and higher fat percentage than back players. Forwards also possessed significantly greater total fat-free mass than backs. All these differences were applicable only to players under 25 years of age. A negative correlation between age and both abdominal and lower extremity fat was found in forward players but not in the backs. Both Yuhasz and Faulkner equations tended to underestimate fat percentage in comparison to Reilly equation. Although Yuhasz equation provided higher systematic error, random error was lower in comparison to Faulkner equation. This study shows the relevance of analyzing and monitoring adiposity in female rugby union players to optimize adaptation to the sports requirements of different playing positions and age.

## 1. Introduction

Rugby union is a highly demanding contact team sport that requires participants to be in exceptionally good physical condition, which includes muscle strength and power, agility, reaction ability, and sprinting speed, among other qualities [1,2]. In addition to frequent heavy impacts, matches combine intermittent periods of high-intensity physical tasks (i.e., sprinting, scrummaging, etc.) with periods of less intensity (i.e., walking, jogging, etc.). In the past, rugby was a male sport, but in recent years, the involvement of women in rugby competitions at different levels has grown markedly all around the world [3]. Rugby requirements for women are also challenging. Using global positioning system tracking technology, the distance covered by elite female rugby union players during a match was found to be 5820 ± 512 m, with a maximum heart rate of more than 90% of their base rate during 50% of the match [4].

In Rugby union, the 15 players on the field assume different playing positions that require specific physical and anthropometric qualities [1,2]. Classically, participants are categorized into forward and back players. Forwards are involved in offensive and defensive collisions, scrums, and ball retention during lineouts and mauls. For better completion of these tasks, forwards usually have greater total body and fat mass than back players [1,5,6,7]. On the contrary, conditioning demands for back players include agility, speed, and reaction ability [8]. To satisfactorily perform their role, backs should be lighter and leaner than forwards and avoid having excess fat.

For more than two decades, differences in anthropometry and body composition between forward and back male players have been extensively addressed in the literature [9,10,11,12,13]. Despite the impact of anthropometry on the performance level of males, there is limited information and lack of consensus on the anthropometric profile of female rugby union players with special reference to adiposity. Some reports describe anthropometric differences between female forward and back rugby union players comparable to those found in male players [7,14,15]. However, other authors did not detect any significant differences among female forwards and backs in both the anthropometric and physical performance measurements [16,17]. Both type of reports, in favor of or against anthropometric and body composition differences between female forwards and backs, only focused on elite rugby union players and did not analyze deeply the impact of adiposity on females. To our knowledge, there are no other studies on sub-elite female rugby union players.

The influence of body composition on physical condition and performance has already been addressed both in male and female players of league rugby, another popular modality of this sport [1,18,19,20]. Specifically, total body fat was negatively correlated with fitness characteristics and performance tests in female rugby league players [15]. The particular and cyclic hormonal environment of women suggests that the monitoring of the body fat component should be relevant in female athletes since there is a close relationship between performance and body fat parameters. Therefore, the aim of this study was to analyze the effect of age (i.e., <21 years, 21–25 years, and >25 years) and players position (i.e., forwards and backs) on body fat distribution (i.e., abdominal fat and lower body fat) and adiposity profile (i.e., sum 7 skinfolds and body fat percentage) in sub-elite female rugby union players. In addition, other objective of the present study was to compare three equations used to estimate the fat mass percentage and analyze the limit of agreement between two of the most widely used equations, Yuhasz and Faulkner vs. Reilly equation, specifically proposed for soccer players [21]. Both rugby and soccer are team sports with similar physiologic demands related to the position played that condition comparable anthropometric characteristics [1,6,22,23]. A deeper knowledge of the body composition profile of these athletes, particularly adiposity, could help to develop specific training programs and physical performance standards for rugby players according to their anthropometric characteristics and the requirements of their playing position. Our hypothesis is that female rugby union forward and back players differ in their adiposity characteristics only during their first years (i.e., younger players) of involvement in competition. After a certain number of years of playing rugby, differences in anthropometry and body composition become less evident.

## 2. Methods

### 2.1. Design

Analytical cross-sectional study of anthropometric data of female rugby union players involved in the sub-elite Spanish National Rugby Championships. Data were recorded at the end of the 2019–2020 season, just prior to the training sessions before the last two matches of the competition.

### 2.2. Participants

A total of 56 female players took part in this study. For analysis and comparison, players were categorized into two groups depending on their position: forwards (*n* = 26) and backs (*n* = 30). The average values of the total sample in terms of age, stature, and body mass were 23.7 ± 6.4 years, 163.5 ± 7.1 cm, and 65.7 ± 10.0 kg, respectively. Regarding age, there were 18 rugby union players under 21 years, 24 players from 21 to 25 years, and 14 players above 25 years. 

All participants had at least two years of experience in rugby training in a structured and organized manner by a sports entity and one year of experience at a professional rugby club. None of the participants received any payment for involvement in the sport. Most of them were university students or generated income through employment outside of rugby. 

At the time of assessment, players trained at least three times a week with sessions lasting over 90 min. These last in-season micro-cycles included a general physical conditioning workout combined with plyometric exercises in the first day of training after the match day. The other two days were devoted to strength and speed training, technical skills, and tactic aspects. We added this information in the manuscript. Competition matches took place on the weekends. The sample was recruited from the players of different rugby teams (UCV, CAU, Les Abelles, and Tecnidex) from the region of Valencia, Spain. Players who did not participate in any activity for more than six weeks due to a sporting injury and those who did not take part in at least six of the last ten competition matches were excluded from the study. 

Both managers of each club and participants were informed of the objectives, procedures, and possible benefits or risks of the study. Informed consent was obtained from all participants as a previous requirement to access the study. The study was conducted in accordance with the Declaration of Helsinki 1961 (reviewed in Edinburgh, 2000) and approved by the Research Ethics Committee of the Catholic University of Valencia (reference: UCV/2019-2020/017). 

### 2.3. Anthropometry and Instruments

All general anthropometric measurements of stature, weight, and thickness of seven skinfolds (biceps, triceps, subscapular, abdominal, suprailiac, thigh, and calf) were taken in accordance with the recommendations from the International Society for the Advancement of Kinanthropometry (ISAK) [24]. To homogenize the hydration status, participants were encouraged to drink at least 1 L of water 30 min before the anthropometric assessment. Each participant’s body mass and stature were measured and recorded with the same equipment, which was regularly calibrated for clinical use. Before a normal training session, all participants were measured and weighed without wearing shoes, with minimal clothing, and with an empty bladder. Regarding precision, stature was measured to the nearest 0.1 cm (SECA 225, SECA, Hamburg, Germany), and weight was measured to the nearest 0.1 kg (SECA 861, SECA, Hamburg, Germany). BMI was calculated as weight (in kilograms) divided by the square of their height (in meters). 

Skinfold thicknesses were measured from the right side of the body with a Holtain Tanner/Whitehouse skinfold caliper (Holtain Ltd., Crymmych, UK). The circumferences of the arms, thighs, and legs were also measured in centimeters. The bi-styloid diameter and both intercondylar diameters in the distal humerus and femur were also measured. All measurements were taken by the same well-trained investigator (ISAK level 2 certified). Each measurement was repeated three times at the same evaluation, and the average value was calculated. The technical measurement error was within the recommended limits by ISAK [24]. 

Body fat percentage were calculated from measurements of two common skinfold equations for the general population, the Yuhasz [25] and the Faulkner equation, which was modified by Slaughter et al. [26]. Since rugby union players have comparable characteristics to soccer players, the Reilly equation, a specific body-fat-predicting equation for soccer players, was also used to test its reliability in our sample [21] (Table 1). Total fat mass was calculated by multiplying the total mass by fat percentage. Fat-free mass was obtained by subtracting from total weight the total fat mass in kilograms. The somatotype components (endomorphy, mesomorphy, and ectomorphy) of each participant were assessed by the Heath–Carter method [27].

### 2.4. Statistical Analyses

All variables were expressed as mean ± standard deviation (SD) and 95% confident interval (CI_95%_). Normal distribution was assessed using Kolmogorov–Smirnov Test. Due to the wide range of the players’ ages, three different age groups were arbitrarily defined for comparison: under 21 years, from 21 to 25, and older than 25. To analyze the effect of age (i.e., under 21 years, 21–25 years, and older than 25 years) and players position (i.e., forwards and backs), a between-groups analysis of variance (ANOVA) was performed. Bonferroni post-hoc corrections were performed to account for type I error. Within age groups, the differences between forwards and backs were analyzed using the nonparametric Mann–Whitney U test, as recommended by the literature for limited samples [28]. To compare the differences between the three equations to estimate fat percentage, a one-way repeated measures ANOVA was performed. The effect sizes were calculated using g Hedges method with the following thresholds: small = 0.20 to 0.49, medium = 0.50 to 0.79, and large >0.80. The associations between anthropometric parameters were described using Pearson correlation coefficient. To account systematic and random error, Bland–Altman plot was used to assess the mean difference and limits of agreement between the Faulkner and Yuhasz equations vs. Reilly equation. Statistical significance was *p* < 0.05. All statistical analyses were performed with the statistical package (Rstudio, v 1.3.959, for MacOS).

### 2.5. Ethical Approval

The study was conducted in accordance with the Declaration of Helsinki 1961 (reviewed in Edinburgh, 2000) and approved by the Research Ethics Committee of the Catholic University of Valencia (reference: UCV/2019-2020/017).

### 2.6. Data Availability

The datasets generated during and/or analyzed during the current study are available from the corresponding author on reasonable request.

## 3. Results

**Figure 1:**
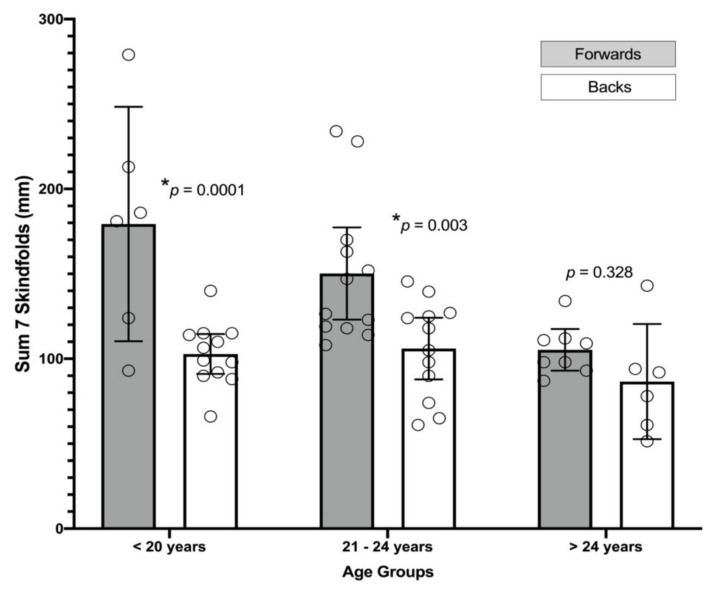
Sum of the seven skinfolds thickness (mm) in the three age-groups regarding players position (i.e., forwards and backs) and age (i.e., <21 years, 21–25 years, and >25 years). Bars represent the mean and 95% of confident interval. * Asterisk represent significant differences (*p* < 0.05).

**Figure 2:**
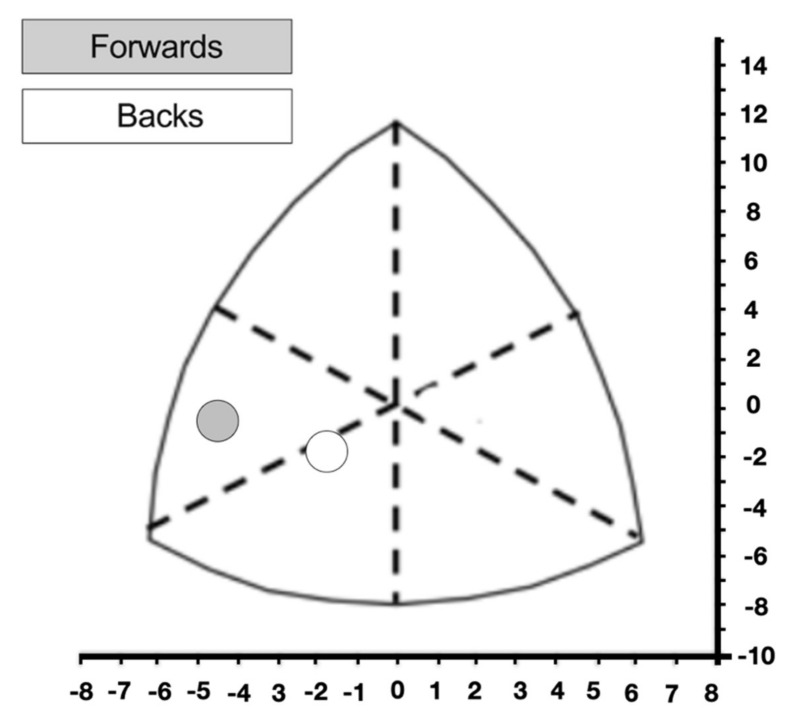
Body composition diagram of the two groups of female rugby players according to Carter and Heath charts.

Table 2 and Table 3 include the general anthropometric characteristics, including adiposity parameters, of the sample. Regarding adiposity profile (i.e., sum of seven skinfolds), the between groups ANOVA showed statistically significant differences in main effect of players position (F _(1,50)_ = 22.43, *p* < 0.001) and *age* (F _(2,30)_ = 6.49, *p* = 0.003) variables. However, no significant differences were found at interaction effect of player position x age (F _(2,50)_ = 2.57, *p* = 0.087). Bonferroni post-hoc comparison revealed a mean difference (MD) and 95% of confident interval (CI_95%_) of 46.47 mm (26.76 to 66.18) between forwards and backs (*p* < 0.001). Regarding age variable, no significant differences were found between <21 years group vs. 21–25 years group (MD = 12.95 mm (−15.01 to 40.93), *p* = 0.770). However, statistically significant differences were found between <21 years group vs. >25 years group and 21–25 years group vs. >25 years group (MD = 45.11 mm (13.24 to 77.03), *p* = 0.003, MD = 32.19 mm (2.96 to 61.52), *p* = 0.027), respectively. See Table 2 and Table 3 and Figure 1 and Figure 2 for more information regarding the other variables.

Forwards had a fat-free mass significantly higher than back players (Table 2). Regarding somatotype, a significantly higher endomorphic component and a lower ectomorphic component were observed in the group of forward players when compared to the backs (*p* < 0.01) (Table 2). Figure 2 displays the body composition diagram of the two groups of female rugby players according to Carter and Heath charts.

**Figure 3:**
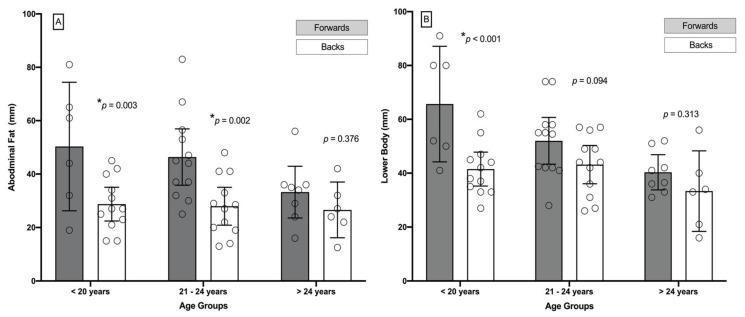
(**A**) Abdominal fat (mm) and (**B**) lower extremity fat (mm) in the three age groups regarding players position (i.e., forwards and backs) and age (i.e., <21 years, 21–25 years, and >25 years). Bars represent the mean and 95% of confident interval. * Asterisk represent significant differences (*p* < 0.05).

Regarding body fat distribution, in abdominal fat, the between groups ANOVA showed statistical significant differences in main effect of players position (F _(1,50)_ = 16.11, *p* < 0.001), but no significant differences were found in age variable (F _(2,30)_ = 6.49, *p* = 0.003). Bonferroni post-hoc comparison revealed a MD and CI_95%_ of 15.56 mm (7.77 to 23.34) between forwards and backs (*p* < 0.001). In relation to age, no significant differences were found at any comparison level (*p* > 0.05). However, statistically significant differences were found in the interaction effect of players position x age: <21 years, forwards vs. backs (MD = 21.58 mm (7.71 to 35.45)) and 21–25 years group, forwards vs. backs (MD = 18.42 mm (7.01 to 29.75)). No significant differences were found in >25 years group, forwards vs. backs (MD = 6.67 mm (−8.83 to 21.65)) (Figure 3).

On the other hand, statistically significant differences were found in the interaction effect of players position x age: <21 years, forwards vs. backs (MD = 24.17 mm (11.43 to 36.90), *p* < 0.001). No significant differences were found between forwards vs. back in 21–25 years group (*p* = 0.094) and >25 years group (*p* = 0.313) (Figure 3).

**Figure 4:**
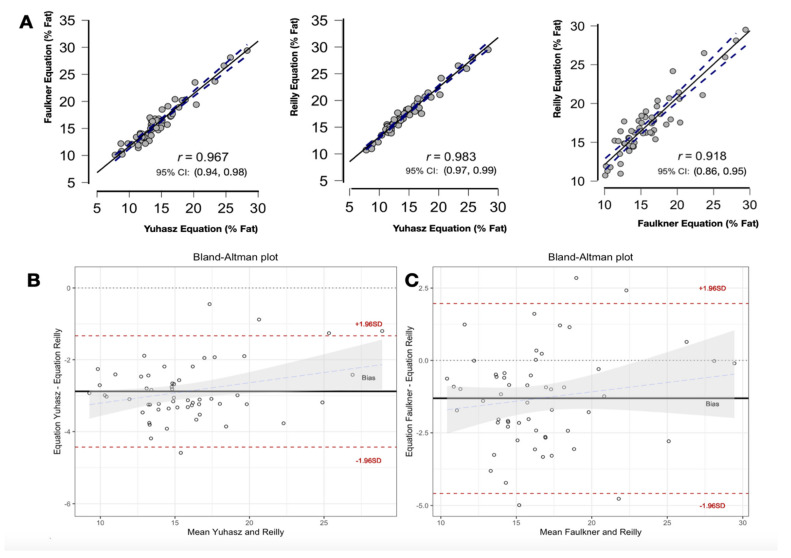
(**A**) Relationships between three fat percentage equations (i.e., Faulkner, Yuhasz, and Reilly). Bland−Altman plot comparing the mean differences and limits of agreement between (**B**) Yuhasz and Reilly equations and (**C**) Faulkner and Reilly equations.

Total fat mass and fat percentages calculated with the different equations were significantly higher in forwards (Table 2). Total fat mass was 56.4% higher in forward than in back players (*p* < 0.001, g Hedges = 1.18 (0.60–1.74)). The Yuhasz equations provided the lowest values of body fat percentage, and the Reilly’s equation yielded the highest values. Differences between these two equations were statistically significant (MD ± SE = 2.9 ± 1.9; *p* = 0.003; g Hedges = 0.72). When three equations were compared, the one-way RM ANOVA showed significant differences (F _(1.16, 63.68)_ = 153, *p* < 0.0001). Bonferroni post-hoc corrections revealed significant differences between Yuhasz vs. Faulkner equations (MD = −1.57% (−1.93 to −1.22), *p* < 0.0001, g Hedges = −1.47 (−1.84 to −1.08)), Yuhasz vs. Reilly equations (MD = −2.88% (−3.14 to −2.62), *p* < 0.0001, g Hedges = −3.62 (−4.34 to −2.89)) and Faulkner vs. Reilly equations (MD = −1.31% (−1.71 to −0.91), *p* < 0.0001, g Hedges = −0.78 (−1.08 to −0.48)). Reilly’s equation, a specific body fat percentage estimation for soccer players, detected the lowest difference in body fat percentage between forwards and backs (3.7 ± 0.9%). Body fat percentage estimations for all three equations were well correlated (see Figure 4). There was also a strong correlation between the fat mass index and the body fat percentage values of the three equations (Faulkner: r = 0.979, *p* > 0.001; Reilly: r = 0.818, *p* > 0.001) for both forwards and backs. Mean differences and limits of agreement between of Yuhasz equation vs. Reilly equation and Faulkner equation vs. Reilly equation correspond to −2.88% (−4.43 to −1.33) and −1.31% (−0.459 to 1.97), respectively, as shown in Figure 4B,C.

## 4. Discussion

The main objective of this study was to analyze the effect of age and players position on body fat distribution and adiposity profile in a sample of sub-elite female rugby union players. In addition, the second objective of this study was to compare three equations used to obtain the fat mass percentage and analyze the limit of agreement between Yuhasz and Faulkner equations vs. Reilly equation. Results obtained showed that there was considerable variation in the anthropometric profile, especially in fat mass and somatotype, which showed a significantly higher body mass, BMI, and adiposity levels in the forwards. In terms of somatotype characteristics, compared to back players, forwards had a predominant endomorphic component with statistical differences. Backs had a significantly lower fat percentage, which mainly defines an ectomorphic somatotype. Fat percentage equations revealed statistical differences between equations. Both Yuhasz and Faulkner equations tended to underestimate fat percentage in comparison to Reilly equation. Although Yuhasz equation provided higher systematic error, random error was more stable. These data provide valuable information in designing specific training programs in accordance with players’ positions and adapting diets to the players’ needs.

To our knowledge, the literature addressing the anthropometry characteristics of female rugby union players is limited to five studies, four of which evaluated elite players (7, 14, 16, 17) and one of which focused on collegiate athletes (22). All these reports come from countries with long traditions in rugby. Nevertheless, the studies covered limited samples of elite players that became fewer when players were discriminated by playing position. The current study described the largest and most unique study so far done on a series of forward and back female rugby union players that focused on sub-elite or non-professional female European players. The current sample revealed lower total body mass, fat mass, and fat percentage in both forwards and backs as compared to data from elite female rugby union players reported in previous studies [14,16,29]. These sub-elite rugby union players had markedly thinner skinfolds than in previous studies, as shown by comparison of the sum of the usual seven skinfold assessments [16,29]. Regarding fat percentage, the estimation for forwards in previous studies varied from a mean of 21.2% (English players) to 30.8% (South Africans). Fat percentage in backs oscillated from 20.2% (English players) to 26.1% (South African players). Our figures were considerably lower: 19.1% and 15.5% for forwards and backs, respectively. Differences found among the six studies could be most likely related to the variety of body composition assessment methods. Even using the DXA, the method shown the lowest variability and greatest accuracy, two of the previously reported studies exhibit quite different results [7,30].

Both forwards and backs in our sample were almost similar in mean age and total body mass to those in Kirby’s study reported in 1993 [14]. Besides the similarities, back players from Spain showed statistically significant lower fat percentages than their English counterparts. The difference cannot be attributed to changes in dietary styles along the last 30 years since body fat percentage in the forward groups was quite similar. In any case, body composition differences between forwards and backs in the current series were coherent with previous research [7,14,16,17,29,30].

The most recent data reporting total body and fat mass in female rugby union players were obtained from a New Zealand cohort of elite players. Although there were no differences in the mean age of players as compared to our series, both New Zealand forwards and backs were significantly heavier (more than 15 kg of mean total mass), had greater fat mass (more than 9 kg of mean fat mass), and showed an estimated higher body fat percentage (more than 6% of the total mass) than sub-elite female players from Spain. These huge differences in the two contemporary series are difficult to explain and may only be attributed to cultural and ethnicity differences between European and Oceanic countries. In fact, different distribution patterns of fat and lean mass have been described in elite male Polynesian rugby union players and in Caucasians [6,12].

The wide range of age of female rugby union players in our sample reflects the current situation of the different teams involved in the Spanish National Women’s Rugby Union Championships. Some players were engaged for several years in competition and had no chance to upgrade to the elite category and continued to play at the sub-elite level. Therefore, players in our sample were discriminated by three different age groups. When the forwards and backs in the three groups were compared, some interesting findings were detected. As hypothesized, forwards’ anthropometric and body composition profiles tended to change with increasing age and progressively showed less total body mass and BMI, and they become thinner (less skinfold thickness and body fat percentage). However, the back players showed almost constant anthropometric and body composition parameters in the three age groups. Among younger players (under 21 years), the differences in body composition profiles between forwards and backs were clear. However, in older players (more than 25 years), there were minimal differences, including lower total body mass and fat mass in backs, between the two groups.

Our findings about the older players conflict with those reported by Nyberg and Penpraze [17], which, in a sample of 19 elite Scottish female rugby union players, found no significant differences between forwards and backs with regards to total body and fat mass. The mean age of their sample was 27.7 ± 7.8 years. In our group of older players (mean age 32.0 ± 7.8 years), forwards still had a heavier body mass (both fat mass and total fat-free mass) than the back players. However, as in the Nyberg and Penpraze series, our forward and back players’ body fat percentages were not significantly different. The difference with respect to Nyberg and Penpraze’s data is that, even in younger participants, our female rugby union players had a much lower body fat percentage. Again, this difference could be likely due in part to the different methods used to estimate body fat percentage. Nyberg and Penpraze used the BOD POD Body Composition System, a method that measures body volume by air displacement to calculate body density and estimate body fat percentage. BOB POD has shown to overestimate body fat percentage as compared to skinfold equations [31]. This fact could confirm our hypothesis regarding the differences between Nyberg and Penpraze’s findings and our current results.

In male rugby union players, total body mass and lean mass increase as their playing level increases [32]. Skinfold thickness and body fat percentage showed the opposite trend and decreased as their playing level improved. According to the current results, female rugby union players revealed a similar tendency. Abdominal, suprailiac, and lower extremity skinfold thickness progressively decreased in the three age-groups analyzed. Consequently, body fat mass also decreased with age. This applied specifically to forwards. Opposite of the previously reported findings in male players [32], total body mass also decreased with age in female forwards. It seems that, in female rugby union players, being heavier has no relevant impact on performance, which has been determined to be the opposite for male players during some of the Rugby World Cups [33,34].

Although this study provides new data to the literature, there are inherent limitations as a direct consequence of the cross-sectional design. As the participants were sub-elite players, many of them did not have a fixed or defined position and were versatile (within their group of forward or back players). Players were only measured once during the season, and it is well documented that anthropometric profiles can change during this time [29]. In addition, no data were collected regarding the eating habits of each player. Furthermore, the estimation of body fat percentage was evaluated through skinfold thickness measurements. It is already known that fat-prediction skinfold equations offer a limited ability to estimate body fat percentage as compared to the standard reference provided by DEXA assessment [35]. Nevertheless, the Reilly equation used in the current study has shown consistency in the fat estimations in sports similar to rugby and was defined as the most suitable equation to evaluate elite male rugby union players [35].

Maintaining optimal body composition is positively related to athletic development in athletes. Depending on the sport, high levels of fat percentage, insufficient levels of fat-free mass, and high BMI can affect performance and health [36]. The research conducted so far agrees that there is no ideal fat value for rugby players. Till et al. [37] conducted a retrospective study in which it was shown that United Kingdom Academy players with lower skinfolds showed long-term progression in their professional sports careers. As a result, evaluating body fat could be the best way to monitor individual changes in diet and/or personalized training as well as other changes [38,39]. In the rugby union case, anthropometric determinations could also be used as a valuable tool for coaches when assessing the profile or position in the game for each player. It seems important to plan training sessions and design a diet in relation to the position of each player on the field to optimize and improve the sporting life of athletes. Finally, an interesting aspect that requires further research is the relation of the somatotype and anaerobic resistance, as the forwards have a higher BMI and must perform strong physical exercises of running short distances at higher speeds.

## 5. Conclusions

This study shows that the adiposity parameters of athletes involved in the sub-elite Spanish National Women’s Rugby Union Championships are related with the player’s position and can vary according to age. Forward players were heavier, had a higher BMI and fat mass, and had thicker skinfolds and a higher fat percentage than back players. Forwards also possessed significantly greater total fat-free mass than backs. All these differences applied only for players under 25 years of age. Female rugby union players included in this study showed thinner skinfolds, lower fat mass, and lesser fat percentage than previously reported for elite female rugby union players versus females of comparable age in other sports. Both Yuhasz and Faulkner equations tended to underestimate fat percentage in comparison to Reilly equation. Although Yuhasz equation provided higher systematic error, random error was lower in comparison to Faulkner equation. These changes could be related to differences in dietary habits and/or the culture of the different countries. This study shows the importance of analyzing and monitoring adiposity in female rugby union players to optimize their adaptation to the requirements of different playing positions.

## Figures and Tables

**Table 1:** Skinfold equation used to estimate body fat percentage (%BF) in females.

Author	Equation	Ref.
Reilly	% BF = 5.174 + (0.124 × thigh) + (0.147 × abdominal) + (0.196 × triceps) + (0.130 × calf)	[21]
Faulkner (Slaughter’s modification)	(Triceps + subscapular < 35 mm) %BF = 1.33 (triceps + subscapular) – 0.013 (triceps + subscapular)^2^ – 2.5 (Triceps + subscapular > 35 mm) %BF = 0.546 (triceps + subscapular) + 9.7	[26]
Yuhasz	%BF = 0.1548 (triceps + subscapular + suprailiac + abdominal + thigh + calf) + 3.580	[27]

**Table 2:** General anthropometric data, skinfold thickness (mm), fat mass, fat-free mass parameters, and body composition profile of the female rugby players included in the study. Differences according to player position are also indicated.

	Whole Equoationsample	Forwards (*n* = 26)	Backs (*n* = 30)	*p*-Value ^§^	g Hedges (CI_95%_)
Age (years)	23.7 ± 6.4	24.8 ± 7.2	22.7 ± 5.5	0.230	0.32 (−0.21 to 0.85)
General anthropometric data					
Stature (cm)	163.5 ± 7.1	164.0 ± 9.3	163.0 ± 4.6	0.603	0.14 (−0.39 to 0.66)
Weight (kg)	65.7 ± 10.0	71.5 ± 10.2	60.6 ± 6.5	0.001 **	1.28 (0.70 to 1.85)
BMI	24.7 ± 4.4	26.8 ± 5.3	22.8 ± 4.4	0.001 **	1.03 (0.46 to 1.58)
Body surface (m^2^)	1.91 ± 0.15	1.99 ± 0.15	1.84 ± 0.11	0.001 **	0.98 (0.42 to 1.54)
Skinfolds					
Triceps (mm)	16.8 ± 6.3	19.5 ± 7.1	14.4 ± 4.3	0.002 **	0.87 (0.32 to 1.42)
Biceps (mm)	8.6 ± 5.1	10.6 ± 6.3	6.8 ± 2.8	0.002 **	0.78 (0.23 to 1.33)
Subscapular (mm)	14.3 ± 7.3	18.1 ± 8.2	10.9 ± 4.1	0.001 **	1.10 (0.53 to 1.66)
Abdominal (mm)	20.3 ± 8.9	24.9 ± 9.1	16.2 ± 6.4	0.001 **	1.10 (0.52 to 1.65)
Suprailiac (mm)	14.8 ± 7.9	18.3 ± 9.4	11.7 ± 4.4	0.001 **	0.90 (0.35 to 1.45)
Thigh (mm)	27.5 ± 8.8	29.6 ± 9.8	25.7 ± 7.6	0.101	0.44 (−0.09 to 0.97)
Leg (mm)	18.1 ± 7.2	21.9 ± 7.6	14.8 ± 4.9	0.000 **	1.11 (0.54 to 1.67)
Fat Mass					
Total Fat Mass (kg)	10.7 ± 4.6	13.3 ± 5.3	8.5 ± 2.2	0.001 **	1.18 (0.60 to 1.74)
FMI	4.1 ± 1.9	5.1 ± 2.3	3.2 ± 0.8	0.001 **	1.10 (0.53 to 1.66)
%BF—Yuhasz	14.3 ± 4.2	16.5 ± 4.7	12.4 ± 2.5	0.001 **	1.08 (0.51 to 1.64)
%BF—Faulkner	15.9 ± 4.2	18.2 ± 4.8	13.9 ± 2.4	0.001 **	1.13 (0.56 to 1.69)
%BF—Reilly	17.2 ± 3.9	19.1 ±4.3	15.5 ± 2.6	0.001 **	1.03 (0.46 to 1.58)
Fat-Free Mass					
Fat-Free Mass (FFM)	58.9 ± 7.5	63.3 ± 7.4	55.3 ± 5.2	0.001 **	1.24 (0.67 to 1.82)
Body composition					
Endomorphy	4.68 ± 1.74	5.56 ± 1.97	3.92 ± 1.05	0.001 **	1.04 (0.48 to 1.60)
Mesomorphy	2.74 ± 1.43	3.13 ± 1.79	2.39 ± 0.91	0.054	0.52 (−0.02 to 1.05)
Ectomorphy	1.71 ± 1.08	1.22 ± 1.23	2.12 ± 0.72	0.001 **	−0.89 (−1.42 to −0.33)

BMI, body mass index; FMI, fat mass index; %BF body fat percentage; CI_95%_, confident interval at 95%; ^§^ Mann−Whitney U test ** (*p* < 0.01).

**Table 3:** General anthropometric data, adiposity parameters, and lean measurements of forward and back players of the three age groups.

	Under 21 Years			From 21 to 25 Years			More than 25 Years		
	Forwards (*n* = 6)	Backs (*n* = 12)	*p*-Value ^§^ (g Hedges)	Forwards (*n* = 12)	Backs (*n* = 12)	*p*-Value ^§^ (g Hedges)	Forwards (*n* = 8)	Backs (*n* = 6)	*p*-Value ^§^ (g Hedges)
General anthropometry									
Body mass (kg)	74.2 ± 11.7	58.9 ± 5.8	0.011 * (1.78)	73.4 ± 11.4	63.4 ± 7.2	0.035 * (1.02)	66.8 ± 5.7	58.5 ± 5.2	0.014 * (1.40)
BMI	31.4 ± 8.1	22.6 ± 1.9	0.031 * (1.75)	26.7 ± 3.9	23.2 ± 2.2	0.026 * (1.08)	23.7 ± 2.1	22.3 ± 1.8	0.302 (0.66)
Fat-Free Mass (kg)	65.0 ± 7.8	53.8 ± 4.8	0.009 ** (1.81)	64.3 ± 8.4	57.5 ± 5.7	0.038 * (0.53)	60.5 ± 56.3	53.7 ± 4.7	0.033 * (1.43)
Adiposity parameters									
Abdominal Fat (mm)	50.3 ± 22.9	28.7 ± 9.9	0.049 * (1.34)	46.4 ± 16.6	27.9 ± 11.1	0.006 * (1.23)	33.2 ± 11.6	26.5 ± 9.9	0.219 (0.57)
Lower Extremity Fat (mm)	65.7 ± 20.5	41.5 ± 9.9	0.015 * (1.63)	52.0 ± 13.6	43.1 ± 11.2	0.183 (0.68)	40.3 ± 7.8	33.3 ± 14.2	0.272 (0.60)
Total Fat mass (kg)	15.9 ± 6.9	8.3 ± 1.8	0.031 * (1.76)	14.4 ± 5.4	9.2 ± 2.6	0.005 ** (1.18)	9.8 ± 1.4	7.8 ± 2.0	0.039 * (1.09)
FMI	6.7 ± 3.0	3.2 ± 0.7	0.025 * (1.93)	5.2 ± 1.9	3.4 ± 0.9	0.004 ** (1.18)	3.5 ± 0.6	3.0 ± 0.8	0.156 (0.65)
%BF—Yuhasz	19.7 ± 6.0	12.5 ± 1.8	0.011 * (1.87)	17.2 ± 4.1	13.0 ± 2.8	0.018 * (1.16)	12.9 ± 1.5	11.2 ± 3.1	0.121 (0.69)
%BF—Slaugther	20.7 ± 6.1	13.9 ± 2.0	0.035 * (1.72)	17.2 ± 4.1	14.3 ± 2.7	0.005 ** (1.30)	12.9 ± 1.5	13.2 ± 2.5	0.121 (0.60)
%BF—Reilly	22.4 ± 5.4	15.7 ± 19	0.015 * (1.86)	19.7 ± 3.9	16.2 ± 2.9	0.033 * (1.00)	15.9 ± 1.2	13.7 ± 2.9	0.053 (1.01)

^§^ Mann−Whitney U test * (*p* < 0.05); ** (*p* < 0.01).

## Data Availability

Data are available upon request to the corresponding author.
